# *CSRP2* transcript levels after consolidation therapy increase prognostic prediction ability in B-cell acute lymphoblastic leukemia

**DOI:** 10.17305/bb.2023.9034

**Published:** 2023-12-01

**Authors:** Lei-Ming Cao, Ya-Lan Zhou, Robert Peter Gale, Ya-Zhen Qin, Li-Xin Wu, Ming-Yue Zhao, Xiao-Su Zhao, Yu-Hong Chen, Yu Wang, Hao Jiang, Qian Jiang, Ying-Jun Chang, Yan-Rong Liu, Lan-Ping Xu, Xiao-Hui Zhang, Xiao-Jun Huang, Guo-Rui Ruan

**Affiliations:** 1 Peking University Institute of Hematology, Peking University People’s Hospital, Beijing Key Laboratory of Hematopoietic Stem Cell Transplantation, Beijing, China; 2 Department of Immunology and Inflammation, Centre for Haematology, Imperial College of Science, Technology and Medicine, London, UK; 3 Academy for Advanced Interdisciplinary Studies, Peking–Tsinghua Center for Life Sciences, Peking University, Beijing, China

**Keywords:** Acute lymphoblastic leukemia (ALL), relapse, measurable residual disease (MRD), cysteine and glycine-rich protein 2 (CSRP2), multi-parameter flow cytometry (MPFC)

## Abstract

Quantification of measurable residual disease (MRD) correlates with the risk of leukemia recurrence in adults with B-cell acute lymphoblastic leukemia (ALL). However, it remains unknown whether collecting data on cysteine and glycine-rich protein 2 (*CSRP2*) transcript levels, after completing the second course of consolidation, improves prognosis prediction accuracy. A total of 204 subjects with B-cell ALL were tested for *CSPR2* transcripts after completing the second course of consolidation using quantitative real-time polymerase chain reaction (qRT-PCR) and divided into high (*N* ═ 32) and low (*N* ═ 172) *CSRP2* expression cohorts. In multivariable analyses, subjects with high expression of *CSRP2* had a higher 5-year cumulative incidence of relapse (CIR) (hazard ratio [HR] ═ 2. 57, 95% confidence interval [CI] 1.38-4.76; *P* ═ 0.003), lower 5-year relapse-free survival (RFS) (HR ═ 3.22, 95% CI 1.75-5.93; *P* < 0.001), and overall survival (OS) (HR ═ 4.59, 95% CI 2.64-7.99; *P* < 0.001) in the whole cohort, as well as in the multi-parameter flow cytometry (MPFC) MRD-negative cohort (for CIR, HR ═ 2.70, 95% CI 1.19-6.12; for RFS, HR ═ 4.37, 95% CI 1.94-9.85; for OS, HR ═ 4.90, 95% CI 2.43-9.90; all *P* < 0.05). Prognostic analysis showed that allogeneic hematopoietic stem cell transplantation (allo-HSCT) could significantly improve the prognosis of patients with high *CSRP2* expression (allo-HSCT vs chemotherapy: 5-year CIR, 52% vs 91%; RFS, 41% vs 9%; OS, 38% vs 20%; all *P* < 0.05). Our data indicate that incorporating data from *CSPR2* transcript levels to the MRD-testing at the end of the second course of consolidation therapy enhances prognosis prediction accuracy in adults with B-cell ALL.

## Introduction

In adults with B-cell acute lymphoblastic leukemia (ALL), which are completing initial therapy, there is a correlation between results of measurable residual disease (MRD) testing and subsequent risk of leukemia recurrence measured as cumulative incidence of relapse (CIR) [1–4]. Most MRD-testing in adults with acute leukemia is based on multi-parameter flow cytometry (MPFC) detection of leukemia-associated immune phenotypes (LAIPs), quantitative polymerase chain reaction (qPCR) amplification-based methods detecting fusion genes, immunoglobulin or T-cell receptor (Ig/TCR) gene rearrangements, or next-generation sequencing (NGS) detecting leukemia-associated mutations [5–8]. The patient scope and sensitivity of each method are different [9–11]. However, some studies have shown that patients who are MRD positive by a PCR-based method but MRD negative by MPFC method are at increased risk for relapse compared with patients who are MRD negative with both methods [12–14]. Consequently, additional methods with higher sensitivity for quantification of MRD and combined monitoring of multiple methods for MRD are needed to improve the ability of prognostic prediction. This would guide the refined risk stratification-based therapy, and ultimately, improve the long-term prognosis of adults with B-cell ALL.

**Figure 1.**
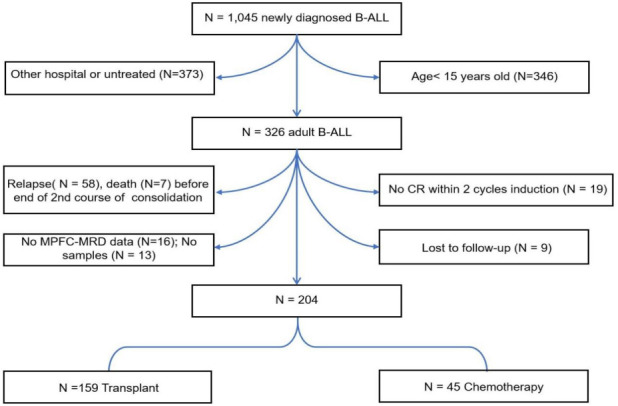
**Consolidated Standards of Reporting Trials (CONSORT) flow diagram.** B-ALL: B-cell acute lymphoblastic leukemia; MPFC-MRD: Multi-parameter flow cytometry-measurable residual disease.

The human cysteine and glycine-rich protein 2 gene (*CSRP2*) encodes the CSRP2 protein consisting of 193 amino acids with a molecular weight of about 21 KD [15]. The CSRP2 protein contains two LIM domains with an inter-domain nuclear localization signal, which may function as a tool for the control of cell growth and differentiation [16, 17]. Hoffmann et al. [18, 19] reported that *CSRP2* expression was significantly upregulated in invasive breast cancer cells and its knockdown significantly reduced the invasive potential of human breast cancer cells in vitro. Tang et al. [20] reported that *CSRP2* expression promoted pulmonary arteries smooth muscle cells (PASMCs) proliferation in vitro. We found that human *CSRP2* transcript levels were upregulated in adults with B-cell ALL at the time of disease diagnosis, which correlated with a higher cumulative incidence relapse (CIR), especially in subjects with normal cytogenetics, and was associated with in vitro drug resistance [21]. We investigated data obtained from 204 consecutive subjects with newly diagnosed B-cell ALL, after completing initial therapy, to determine if quantifying *CSRP2* expression could be used to predict relapse. We found that MRD-testing at the end of the second course of consolidation therapy by *CSRP2* transcript levels was an independent predictor of relapse and survival in multivariable analyses in subjects receiving subsequent maintenance chemotherapy or an allotransplant.

## Materials and methods

### Subjects

A total of 1045 people with newly-diagnosed B-cell ALL were found at the Peking University Institute of Hematology from 2012 to 2019. Subjects younger than 15 years (*N* ═ 346) and/or subjects who received their initial therapy elsewhere (*N* ═ 373) were excluded from the study. In addition, 19 other subjects who did not achieve a complete hematological remission after 2 courses of induction chemotherapy were also excluded. Sixty-five of the remaining 307 subjects were excluded because of the relapse (*N* ═ 58) or death (*N* ═ 7) before completing the second consolidation therapy course. Another 9 subjects were excluded due to the discontinued follow-up, as well as 13 subjects with no available samples and 16 without complete MPFC data. The remaining 204 consecutive subjects, in the range of 15–69 years, were enrolled (Consolidated Standards of Reporting Trials diagram; Figure 1).

Diagnosis of B-cell ALL was based on World Health Organisation (WHO) 2016 criteria [22]. Hematological complete remission was defined as bone marrow lymphoblasts < 5%, granulocyte concentration > 1.0 × 10E^9^/L, platelet concentration > 100 × 10E^9^/L, hemoglobin concentration > 100 g/L, no extra-medullary leukemia and no change in these criteria for > 1 month. Relapse was defined as the number of bone marrow lymphoblasts ≥ 5% at any site in subjects achieving hematological complete remission.

### Therapy for the treatment of ALL

For subjects without *BCR::ABL1* fusion gene, the main induction chemotherapy was cyclophosphamide, vindesine, daunorubicin, and prednisone (CODP) with (*N* ═ 52) or without (*N* ═ 66) L-asparaginase. For *BCR::ABL1* positive subjects, in the year 2017 and before, the main induction chemotherapy was CODP plus tyrosine kinase inhibitor (TKI) (*N* ═ 62) (imatinib, *N* ═ 46; dasatinib, *N* ═ 14; ponatinib, *N* ═ 2). The used therapy for the rest of the subjects was vindesine plus prednisone (VP) plus TKI (*N* ═ 8) (imatinib, *N* ═ 6; dasatinib, *N* ═ 2). After the year of 2017, the main induction chemotherapy was VP plus TKI (*N* ═ 13) (imatinib, *N* ═ 6; dasatinib, *N* ═ 5; ponatinib, *N* ═ 2), and the rest was CODP plus imatinib (*N* ═ 3). Subjects achieving a hematological complete remission received consolidation chemotherapy for ≥ 2 courses of hyper-CVAD (B), hyper-CVAD (A) (cyclophosphamide, vindesine, epirubicin, and dexamethasone) or CAM (cyclophosphamide, cytarabine, and 6-mercaptopurine). After the completion of the second course of consolidation therapy, subjects received a transplant if an appropriate HLA-identical or -matched donor was available. Other subjects were given a choice between maintenance chemotherapy with methotrexate, 6-mercaptopurine, vindesine, prednisone for the period of 2–2.5 years or HLA-haplotype-matched transplant [23]. The details about the therapies are displayed in Table S1. All subjects received central nervous system prophylaxis with intrathecal methotrexate and/or cytarabine for ≥ 8 doses during induction chemotherapy and consolidation therapy.

### RNA extraction and synthesis of cellular DNA (cDNA)

Mononuclear cells were isolated from bone marrow samples by Ficoll-Hypaque density gradient centrifugation (Solarbio Technology, Beijing, China) at diagnosis and after completing the second course of consolidation. Total cellular RNA was extracted using Trizol^®^ kits (Invitrogen, Carlsbad, CA, USA) according to the manufacturer’s instructions. Complementary DNA (cDNA) synthesis was done as described [24].

### Measurement of relative *CSRP2* transcript levels by qRT-PCR

Bone marrow samples at the end of the second course of consolidation therapy were analyzed by the relative transcript levels of *CSRP2*. TaqMan^®^ quantification was done using the ABI PRISM^®^ 7500 FAST Sequence Detection System (Applied Biosystems, Foster City, CA, USA) with *ABL1* as an internal control. The primer and probe sequence of *CSRP2* and *ABL1* were designed using Primer-Express software (Applied Biosystems) and displayed in Table S2. qRT-PCR was done as described [24]. *CSRP2* and *ABL1* copy numbers were calculated as described in our previously published paper [21].

### Immune phenotype, cytogenetic and molecular analyses

Bone marrow samples collected at diagnosis and after completing the second course of consolidation therapy were analyzed for leukemia-associated aberrant immune phenotypes (LAIPs) using standard eight-color MPFC. In most B-cell ALL cases, CD34-FITC/CD10-PE/CD45-perCP/CD19-APC and CD22-FITC/CD20PE/CD45-perCP/CD19-APC or CD58-FITC/CD123-PE/ and CD45-perCP/CD19-APC antibody combinations were sufficient to identify leukemic cells. A different-from-normal approach was used when a LAIP could not be assigned. A positive MPFC MRD-testing is defined as > 0.01% [25–27]. Cytogenetic analyses were done by G-banding [28]. *WT1* and *BCR::ABL1* transcripts and *KMT2A* fusion genes (*KMT2A-AF4, KMT2A-AF9, KMT2A-AF1p,* and *KMT2A-AF1q*) fusion transcripts were detected by TaqMan-based quantitative real-time polymerase chain reaction (qRT-PCR) as described [29]. The *BCR::ABL1* transcripts were analyzed by qRT-PCR with a sensitivity of 10E-6. *IKZF1* deletions were detected using multiplex qRT-PCR, multiplex fluorescent PCR, and sequence analysis [30].

### Ethical statement

The study was approved by the Ethics Committee of Peking University People’s Hospital and all subjects have signed written informed consent consistent with the principles of the Helsinki Declaration. This trial has been registered in the Beijing Municipal Health Bureau Registration N 2007-1007 and in the Chinese Clinical Trial Registry [ChiCTR-OCH-10000940 and ChiCTR-OPC-14005546].

### Statistical analysis

CIR was calculated as the interval from completing the second consolidation course to relapse, last follow-up, or withdrawal of consent. Cumulative incidences were estimated for relapse to accommodate competing risks. Relapse-free survival (RFS) was calculated from the completion of the second consolidation course to relapse, last follow-up, or withdrawal of consent. Overall survival (OS) was calculated as the interval from completing the second consolidation course to death, last follow-up, or withdrawal of consent. The threshold value to divide *CSRP2* transcript levels into high and low cohorts was determined by the receiver operating characteristic (ROC) curve based on CIR data. Student’s *t*-test and Mann–Whitney *U* tests were used to analyze normal continuous variables and non-normal continuous variables. Pearson chi-square or Fisher exact tests were used to evaluate categorical co-variates. The Bonferroni procedure was used to perform multiple comparisons. Survival functions were estimated by the Kaplan–Meier method and compared by the log-rank test. A Cox proportional hazard regression model was used to determine correlations among MRD defined by *CSRP2* transcript level, RFS, and OS. A competing risk model was used to determine associations between *CSRP2* transcript levels and CIR. Co-variates with *P <* 0.20 in univariable analyses were included in multivariable analysis. *P* < 0.05 in a 2-sided test was considered statistically significant. Analyses were performed by SAS version 9.4 (SAS Institute Inc., Cary, NC, USA), Graphpad Prism^TM^ 9.0.0 (San Diego, California, USA), and R software package (version 4.0.3; http://www.r-project.org). A negative *BCR::ABL1* at the end of the second course of consolidation therapy was defined as an individual ≥ 3 log reduction from the *BCR::ABL1* transcript level at diagnosis [31].

## Results

### Subject- and disease-related co-variates and outcomes

We studied 204 consecutive subjects who achieved a hematological complete remission after 1 (*N* ═ 192) or 2 (*N* ═ 12) courses of induction chemotherapy and remained in remission after 2 courses of consolidation therapy. In a preliminary analysis, there were no statistically significant differences in CIR, RFS, or OS between subjects requiring one or two induction chemotherapy courses to achieve a hematological complete remission and these cohorts were combined in subsequent analyses. The median follow-up of survivors was 31 months (interquartile range [IQR] 17–56 months). The median age of subjects was 34 years (IQR 24–46 years), and 111 were male. A total of 159 subjects (78%) received allotransplant, with a median of 3 months (IQR 2–4 months) after completing the second consolidation course. Forty-five others received maintenance chemotherapy only. Seventy subjects had a hematological relapse. The median interval from completing the second consolidation course to relapse was 13 months (IQR 5–25 months). Fifty-five subjects (27%) died of relapse (*N* ═ 39) or transplant-related mortality (*N* ═ 16). Details are displayed in Table 1.

**Table 1:** Subject- and leukemia-related co-variates

**Variables**	**Total, *N ═* 204**	**Low *CSRP2, N* ═ 172**	**High *CSRP2, N* ═ 32**	***P* value**
Male, *N* (%)	111 (54)	91 (53)	20 (63)	0.42
Age (years)^1^				0.20
Median (range)	34 (15–69)	34 (15–69)	36 (16–59)	
Hemoglobin (g/L)^1^				0.47
Median (range)	93 (29–164)	93 (29–160)	87 (50–164)	
WBC^1^ ≥ 30×10E+9/L, *N* (%)	71 (35)	53 (31)	18 (56)	0.01
Platelets (×10E+9/L)^1^				0.20
Median (range)	59 (3–352)	61 (3–352)	48 (5–311)	
Bone marrow blasts^1^ (%)				0.80
Median (range)	88 (16–99)	89 (16–99)	87 (55–98)	
Immune phenotype^1^, *N* (%)				0.45
Common	155 (76)	131 (76)	24 (75)	
Pre-B	22 (11)	20 (12)	2 (6)	
Pro-B	27 (13)	21 (12)	6 (19)	
Cytogenetics^1^, *N* (%)				
Normal	67 (33)	59 (34)	8 (25)	0.30
t(9;22)(q34;q11)	66 (32)	56 (33)	10 (31)	0.88
t(1;19)(q23;p13)	5	5	0	–
Hyperdiploid	2	2	0	–
Hypodiploid	1	1	0	–
* KMT2A* rearrangement^2^	8 (4)	4 (2)	4 (13)	0.03
* BCR::ABL1*^2^ positive	32 (16)	25 (15)	7 (22)	0.43
* IKZF1* deletion^2^	19 (9)	15 (9)	4 (13)	0.73
MPFC-MRD^2^ positive	57 (28)	40 (23)	17 (53)	0.001
Post-consolidation therapy, *N* (%)				0.11
Transplant	159 (78)	138 (80)	21 (65.6)	
Chemotherapy maintenance	45 (22)	34 (20)	11 (34)	

^1^Detected at diagnosis: ^2^Detected at the end of the second course of consolidation therapy; MPFC-MRD: Multi-parameter flow cytometry measurable residual disease; *CSRP2:* Cysteine and glycine-rich protein 2; WBC: White blood count.

### Serial determinations of *CSRP2* transcript levels

**Figure 2.**
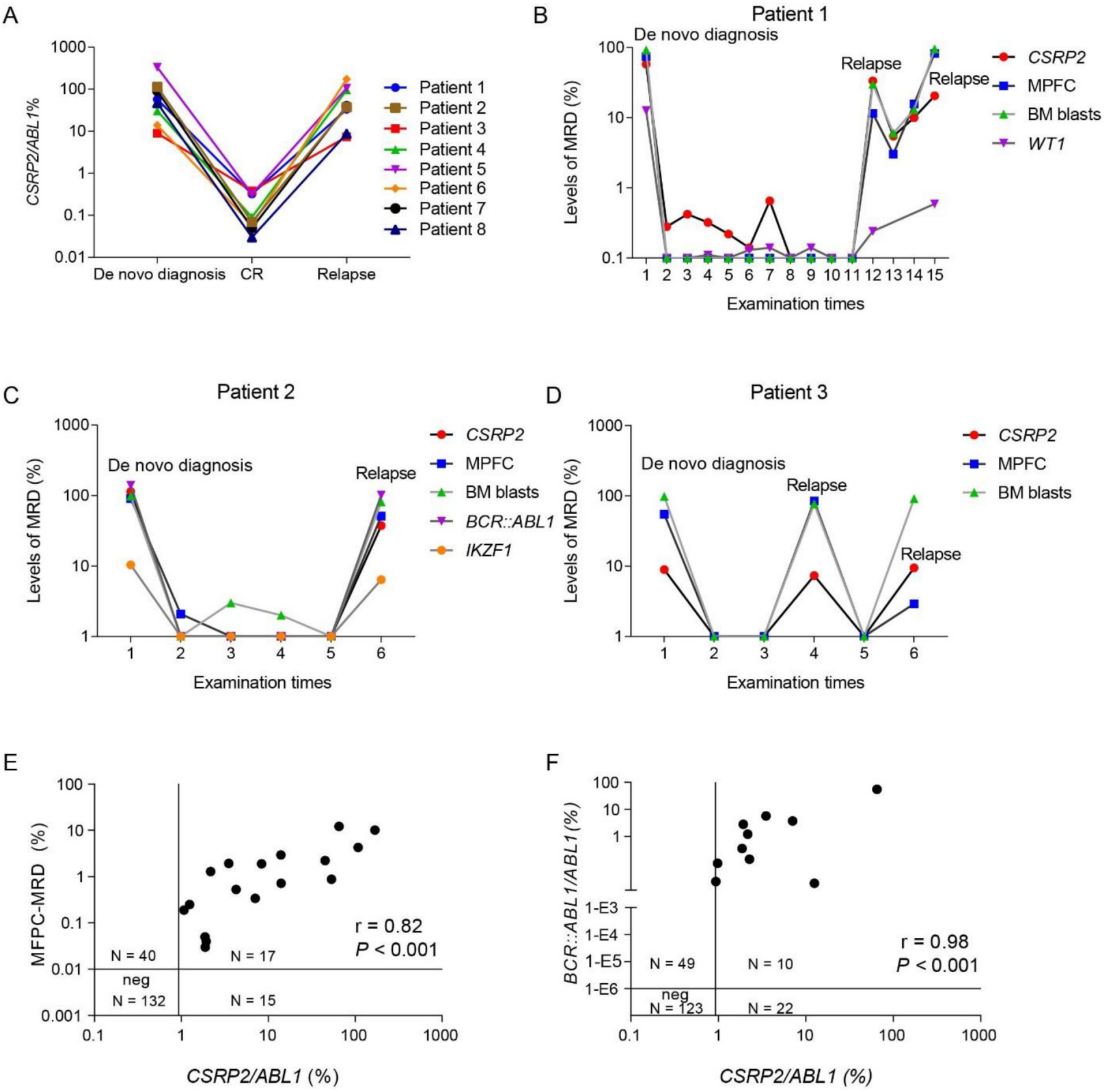
**Correlation of *CSRP2* transcript levels with clinical course and other MRD-tests**. (A) Correlation between *CSRP2* transcript level and clinical course in eight subjects at diagnosis, in complete hematological remission and at relapse; (B–D) Dynamic *CSRP2* transcript levels in three subjects with long-term follow-up; (E and F) *CSRP2* transcript levels with MPFC-MRD and *BCR::ABL1* at the end of the second course of consolidation therapy. BM blasts: Bone marrow blasts; *CSRP2:* Cysteine and glycine-rich protein 2; MRD: Measurable residual disease; MPFC: Multi-parameter flow cytometry.

We studied serial determinations of *CSRP2* transcript levels in bone marrow samples from eight subjects at diagnosis, in complete hematological remission, and at relapse. *CSRP2* transcript levels in complete hematological remission were significantly lower compared with diagnosis or relapse samples (Figure 2A). In 3 subjects with long-term follow-up, we compared the results of *CSRP2* testing with other MRD assays, including MPFC (*N* ═ 3), *WT1* and *BCR::ABL1* transcript levels (*N* ═ 1), and *IKZF1* deletion (*N* ═ 1; Figures 2B–2D). *CSRP2* transcript levels correlated well with clinical courses, as well as with other evaluated assays.

### *CSRP2* transcript levels after consolidation

Subjects were divided into high (*N* ═ 32) and low (*N* ═ 172) cohorts based on a *CSRP2* transcript at the end of the second course of consolidation therapy ≥ or < 0.93 percent of *ABL1* transcript value determined by ROC curve based on CIR data. Clinical and laboratory co-variates were similar between cohorts except for white blood count (WBC) at diagnosis and MPFC-testing positivity or *KMT2A* rearrangement at the end of the second course of consolidation therapy (which was scored as MRD-positive; all *P* values < 0.05; Table 1).

*CSRP2* transcript levels were analyzed for correlations with results of MPFC- and *BCR::ABL1-*testing. The MPFC testing was positive in 17 subjects (53%) in the high *CSRP2* transcript cohort vs 40 (23%; *P* ═ 0.001) in the low *CSRP2* transcript cohort. *BCR::ABL1* transcripts were detected in 7 subjects (22%) in the high *CSRP2* transcript cohort vs 15 (15%; *P* ═ 0.43) in the low *CSRP2* transcript cohort. One hundred and thirty-two (132/172, 77%) subjects were negative for MRD by MPFC-testing in the low *CSRP2* transcript cohort and 17 (17/32, 53%) were positive for MRD by MPFC-testing in the high *CSRP2* transcript cohort with a concordance of 73% (*r* ═ 0.82; *P* < 0.001; Figure 2E). In the high *CSRP2* transcript cohort, 2 of 12 subjects who were *BCR::ABL1*-positive at diagnosis became negative at the end of the second course of consolidation therapy compared with 26 of 74 in the low *CSRP2* transcript cohort. Concordance for MRD-testing between *BCR::ABL1* and *CSRP2* transcripts was 65% (*r* ═ 0.98; *P <* 0.001; Figure 2F).

**Figure 3.**
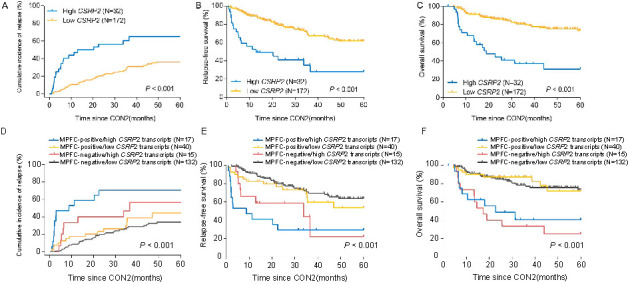
**Outcomes in subjects based on *CSRP2* cohort and MPFC-testing result.** CIR (A), RFS (B), and OS (C) were compared between subjects with high or low *CSRP2* transcript levels; CIR (D), RFS (E), and OS (F) of subjects in cohort-1 (MPFC-positive/high *CSRP2* transcripts), cohort-2 (MPFC-positive/low *CSRP2* transcripts), cohort-3 (MPFC-negative/high *CSRP2* transcripts), and cohort-4 (MPFC-negative/low *CSRP2* transcripts). MPFC combined with *CSRP2* transcript level at the end of the second course of consolidation therapy better stratified patients and multiple comparisons based on the Bonferroni procedure were performed (CIR, all *P* > 0.05, cohort-1 vs cohort-2, *P* ═ 0.02, cohort-1 vs cohort-4, *P* < 0.001; RFS, all *P* > 0.05, cohort-1 vs cohort-2, *P* ═ 0.02, cohort-1 vs cohort-4, *P* < 0.001, cohort-3 vs cohort-4, *P* ═ 0.01; OS, all *P* < 0.05, cohort-1 vs cohort-3, *P* ═ 1.00, cohort-2 vs cohort-4, *P* ═ 1.00). CON2: The second course of consolidation therapy; *CSRP2*: Cysteine and glycine-rich protein 2; MPFC: Multi-parameter flow cytometry; CIR: Cumulative incidence of relapse; RFS: Relapse-free survival; OS: Overall survival.

Twenty subjects (63%) in the high *CSRP2* transcript level cohort relapsed compared with 50 (29%) in the low *CSRP2* transcript level cohort (*P* ═ 0.001). We found that subjects with high *CSRP2* transcript level had a higher CIR, worse RFS, and OS compared with those with low *CSRP2* transcript levels. The 5-year CIRs were 65% (95% confidence interval [CI] 44%, 80%) vs 36% (95% CI 28%, 45%) in the high vs low *CSRP2* transcript level cohorts (hazard ratio [HR] ═ 3.10 [95% CI 1.76, 5.45]; *P* < 0.001; Figure 3A). The 5-year RFS rates were 28% (95% CI 11%, 48%) vs 62% (95% CI 52%, 70%; HR ═ 3.50 [95% CI 1.62, 7.58]; *P* < 0.001); Figure 3B). The 5-year OS was 31% (95% CI 14%, 49%) vs 73% (95% CI 64%, 81%); HR ═ 4.36 (95% CI 1.91, 9.96); *P* < 0.001; Figure 3C).

In multivariable analyses, WBC ≥ 30×10E+9/L at diagnosis (HR ═ 1.82 [95% CI 1.05, 3.17]; *P* ═ 0.03), high *CSRP2* transcript level (HR ═ 2.57 [95% CI 1.38, 4.76]; *P* ═ 0.003), maintenance chemotherapy vs transplant (HR ═ 5.56 [95% CI 3.23, 10.00]; *P* < 0.001), *KMT2A* rearrangement at the end of the second course of consolidation therapy (HR ═ 3.10 [95% CI 1.19, 8.07], *P* ═ 0.02), and a positive MPFC-testing (HR ═ 1.96 [95% CI 1.17, 3.29]; *P* ═ 0.01) were independently associated with higher 5-year CIR. These covariates were also significantly associated with worse 5-year RFS (HR 1.74 [95% CI 1.05, 2.90], *P* ═ 0.03; HR ═ 3.22 [95% CI 1.75, 5.93], *P* < 0.001; HR ═ 5.56 [95% CI 3.33, 10.00], *P* < 0.001; HR ═ 2.76 [95% CI 1.21, 6.33], *P* ═ 0.02; and HR ═ 1.74 [95% CI 1.05, 2.90], *P* ═ 0.04; respectively). Only high *CSRP2* transcript levels at the end of the second consolidation course and post-consolidation maintenance chemotherapy were associated with worse OS (HR ═ 4.59 [95% CI 2.64, 7.99], *P* < 0.001; HR ═ 2.13 [95% CI 1.19, 3.70], *P* ═ 0.01; Table 2).

To determine whether combining data from results of MPFC testing and *CSRP2* transcript levels improves CIR prediction accuracy, we divided subjects into 4 cohorts: (1) MPFC-positive/high *CSRP2* transcripts (*N* ═ 17); (2) MPFC-positive/low *CSPR2* transcripts (*N* ═ 40); (3) MPFC-negative/high *CSRP2* transcripts (*N* ═ 15); and (4) MPFC-negative/low *CSRP2* transcripts (*N* ═ 132). The combined test had good value for predicting CIR (71% [95% CI 41%, 87%] vs 44% [95% CI 25%, 63%] vs 57% [95% CI 23%, 80%] vs 34% [95% CI 25%, 43%]; *P* < 0.001; Figure 3D). Similar trends were detected for RFS (RFS, 29% [95% CI 11%, 51%] vs 54% [95% CI 33%, 71%] vs 22% [95% CI 1%, 58%] vs 64% [95% CI 53%, 73%], *P* < 0.001; Figure 3E). In addition, OS also showed similar trend as CIR and RFS (OS, 41% [95% CI: 16%, 64%] vs 72% [95% CI 50%, 86%] vs 25% [95% CI 7%, 49%] vs 73% [95% CI 62%, 81%], *P* < 0.001; Figure 3F).

**Table 2:** Multivariable analyses of 5-year CIR, RFS, and OS

**Outcomes**	**HR (95% CI)**	***P* value**
*CIR*		
WBC^1^ (≥ 30 vs < 30×10E+9/L)	1.82 (1.05, 3.17)	0.03
*CSRP2*^2^ (high vs low)	2.57 (1.38, 4.76)	0.003
Chemotherapy maintenance	5.56 (3.23, 10.00)	<0.001
*KMT2A* rearrangement^2^ (positive vs negative)	3.10 (1.19, 8.07)	0.02
MPFC-MRD^2^ (positive vs negative)	1.96 (1.17, 3.29)	0.01
*RFS*		
WBC^1^ (≥ 30 vs < 30×10E+9/L)	1.74 (1.05, 2.90)	0.03
*CSRP2^2^* (high vs low)	3.22 (1.75, 5.93)	<0.001
Chemotherapy maintenance	5.56 (3.33, 10.00)	<0.001
*KMT2A* rearrangement^2^ (positive vs negative)	2.76 (1.21, 6.33)	0.02
MPFC-MRD^2^ (positive vs negative)	1.74 (1.05, 2.90)	0.04
*OS*		
*CSRP2^2^* (high vs low)	4.59 (2.64, 7.99)	<0.001
Chemotherapy maintenance	2.13 (1.19, 3.70)	0.01

^1^Detected at diagnosis: ^2^Detected at the end of the second course of consolidation therapy; CIR: Cumulative incidence of relapse; RFS: Relapse-free survival; OS: Overall survival; HR: Hazard ratio; CI: Confidence interval; MPFC-MRD: Multi-parameter flow cytometry-measurable residual disease; *CSRP2:* Cysteine and glycine-rich protein 2; WBC: White blood count.

### *CSRP2* transcript levels and outcomes in MPFC-MRD-negative patients

**Figure 4.**
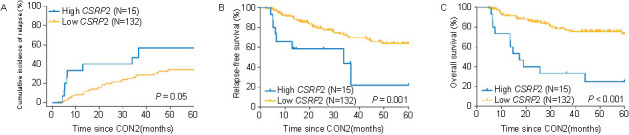
**Outcomes of *CSRP2* transcript levels in negative MPFC-MRD subjects at the end of the second course of consolidation therapy.** Cumulative incidence of relapse (A), relapse-free survival (B), and overall survival (C) were compared between subjects with high or low *CSRP2* transcript levels. CON2: The second course of consolidation therapy; MPFC-MRD: Multi-parameter flow cytometry measurable residual disease; *CSRP2*: Cysteine and glycine-rich protein 2.

In addition, we analyzed 147 subjects with a negative MPFC testing. More than half (8/15, 53%) of the MPFC-negative/high *CSRP2* group experienced recurrence. On the other hand, in the MPFC-negative/low *CSRP2* group (*N* ═ 132), the relapse rate was relatively low (36/132, 27%). The 5-year CIR of the high *CSRP2* transcript cohort was 57% (95% CI 23%, 80%) compared with 34% (95% CI 25%, 43%) in the low *CSRP2* transcript cohort (HR ═ 2.38 [95% CI 1.04, 5.45]; *P* ═ 0.05; Figure 4A). The 5-year RFSs were 22% (95% CI 1%, 58%) vs 64% (95% CI 53%, 73%); (HR ═ 3.31 [95% CI 0.98, 11.15]; *P* ═ 0.001; Figure 4B). The 5-year OSs were 25% (95% CI 7%, 49%) vs 73% (95% CI 62%, 81%); (HR ═ 4.87 [95% CI 1.48, 16.03]; *P* < 0.001; Figure 4C). In multivariable analyses, high *CSRP2* transcript level, chemotherapy maintenance rather than a transplant, and positive *KMT2A* rearrangement, all at the end of the second course of consolidation therapy were significantly correlated with higher CIR (HR ═ 2.70 [95% CI 1.19, 6.12], *P* ═ 0.02; HR ═ 2.94 [95% CI 1.52, 5.56], *P* ═ 0.002; HR ═ 7.16 [95% CI 3.88, 13.19], *P* < 0.001, respectively) and worse RFS (HR ═ 4.37 [95% CI 1.94, 9.85], *P* < 0.001; HR ═ 3.13 [95% CI 1.61, 5.88], *P* ═ 0.001; HR ═ 6.60 [95% CI 2.69, 16.17], *P* < 0.001, respectively). Only a high *CSRP2* transcript level was significantly associated with worse OS (HR ═ 4.90 [95% CI 2.43, 9.90], *P* < 0.001; Table 3).

**Figure 5.**
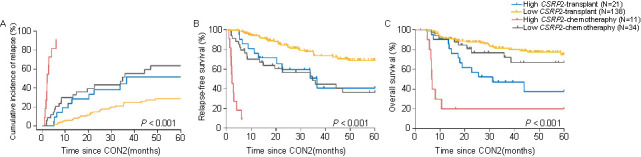
**Correlations between outcomes and post-consolidation therapy and *CSRP2* transcript levels.** (A) Cumulative incidence of relapse; (B) Relapse-free survival; (C) Overall survival. CON2: The second course of consolidation therapy; *CSRP2*: Cysteine and glycine-rich protein 2.

### Impact of *CSRP2* on outcomes in the transplant and chemotherapy cohorts

Forty-one transplant recipients (26%) and 29 maintenance chemotherapy recipients (64%) relapsed (*P* < 0.001). Next, we stratified subjects by post-consolidation therapy into 4 cohorts: (1) transplant/high *CSRP2* transcript level (*N* ═ 21); (2) transplant/low *CSRP2* transcript level (*N* ═ 138); (3) chemotherapy/high *CSRP2* transcript level (*N* ═ 11); (4) chemotherapy/low *CSRP2* transcript level (*N* ═ 34). These cohorts had significantly different CIRs of 52% ([95% CI 26%, 72%], 29% [95% CI 20%, 38%], 91% [95% CI 29%, 99%], and 63% [95% CI 42%, 79%], respectively; *P* < 0.001; Figure 5A). The 5-year RFS differed (41% [95% CI 16%, 65%] vs 69% [95% CI 58%, 76%] vs 9% [95% CI 1%, 42%] vs 37% [95% CI 19%, 54%]; *P* < 0.001; Figure 5B). In addition, 5-year OS also differed (38% [95% CI 15%, 60%] vs 75% [95% CI 64%, 83%] vs 20% [95% CI 3%, 47%] vs 67% [95% CI 45%, 82%]; *P* < 0.001; Figure 5C).

**Table 3:** Multivariable analyses of 5-year CIR, RFS, and OS in MPFC-negative subjects

**Outcomes**	**HR (95% CI)**	***P* value**
*CIR*		
*CSRP2^1^* (high vs low)	2.70 (1.19, 6.12)	0.02
Chemotherapy maintenance	2.94 (1.52, 5.56)	0.002
*KMT2A* rearrangement^2^(positive vs negative)	7.16 (3.88, 13.19)	<0.001
*RFS*		
*CSRP2^1^* (high vs low)	4.37 (1.94, 9.85)	<0.001
Chemotherapy maintenance	3.13 (1.61, 5.88)	0.001
*KMT2A* rearrangement^1^ (positive vs negative)	6.60 (2.69, 16.17)	<0.001
*OS*		
*CSRP2^1^* (high vs low)	4.90 (2.43, 9.90)	<0.001

^1^Detected at the end of the second course of consolidation therapy. CIR: Cumulative incidence of relapse; RFS: Relapse-free survival; OS: Overall survival; HR: Hazard ratio; CI: Confidence interval; MPFC: Multi-parameter flow cytometry; *CSRP2:* Cysteine and glycine-rich protein 2.

## Discussion

Our data indicate that *CSRP2* transcript levels, after the second course of consolidation therapy, are independently associated with 5-year CIR, RFS, and OS in adults with B-cell ALL receiving maintenance chemotherapy or an allotransplant. Providing data of *CSRP2* transcript levels, in addition to results of MPFC-testing for MRD, improved relapse and survival prediction accuracy.

Included subjects with B-cell ALL were relatively young, with a median age of 34 years (IQR, 24–46 years), which probably reflected the transplant-related selection bias. The young age distribution may also explain the relatively few subjects with the *BCR::ABL1* fusion gene.

There are several reasons why high *CSRP2* level expression, after completing the second course of consolidation therapy, might correlate with an increased CIR. One is the indication of more residual leukemia cells compared with subjects with low *CSRP2* expression. The second possibility relates to the mechanism of action of *CSRP2* which favors cell proliferation, promotes cell-cycle progression, and inhibits apoptosis [18–21]. These explanations are not mutually exclusive.

There are several important limitations to our study. First, it is a retrospective study. Second, sample size is relatively small and there was no external validation cohort to test the threshold value of *CSRP2* transcript level. Third, our comparator MRD evaluation was MPFC rather than a molecular method, such as *IGH* rearrangement by NGS. Fourth, the relatively few subjects with *BCR::ABL1* fusion genes limited the power of our analyses. Fifth, the assignment to post-consolidation therapy was not random. These limitations require further validation of our conclusions in larger prospective studies.

## Conclusion

In summary, our data indicate that providing data about *C*S*PR2* transcript level, in addition to the results of MPFC MRD-testing at the end of conventional therapy, improves relapse and survival prediction accuracy in adults with B-cell ALL regardless of subsequent therapy.

## Supplemental Data

**Table S1:** Details of therapy used for the treatment of subjects with acute lymphoblastic leukemia

**Therapy**	**Dose and schedules**
*CODP*	
Cyclophosphamide	750 mg/mE+2, day 1
Vindesine	4 mg, days 1, 8, 15 and 22
Daunorubicin	40–45 mg/mE+2, days 1 to 3
Prednisone	1 mg/kg/day
*CODP + L*	
Cyclophosphamide	750 mg/mE+2, day 1
Vindesine	4 mg, days 1, 8, 15 and 22
Daunorubicin	40–45 mg/mE+2, days 1 to 3
Prednisone	1 mg/kg/day
L-asparaginase	10,000 U - days 15 to 24 / 3,750 U on day 15
*VP*	
Vindesine	1.4 mg/mE+2, weekly
Prednisone	1 mg/kg/day
*Hyper-CVAD (A)*	
Cyclophosphamide	300 mg/mE+2, once every 12 h, day 1 to 3
Vindesine	4 mg, days 4 and 11
Epirubicin	60 mg /mE+2, day 4
Dexamethasone	40 mg, days 1 to 4, and 11 to 14
Methotrexate	1–1.5 g/mE+2/day, day 1
Asparaginase	3,750 U on day 3
*Hyper-CVA (B) (modified)*	
Methotrexate	1 g/mE+2, day 1
Cytosine arabinoside	1 g/mE+2, once every 12 h, days 2 to 3
*FLAG*	
Fludarabine	25 mg/mE+2, days 1 to 5
Cytarabine	1 g/mE+2, days1 to 5
Granulocyte colony- stimulating factor (G-CSF)	5 µg/kg, days 1 to 5
*CAM*	
Cyclophosphamide	1 g/mE+2/d, day 1
Cytarabine	100 mg/mE+2/d, days 1 to 7
6-mercaptopurine	50 mg/mE+2/d, days 1 to 7
*Methotrexate and asparginase*	
Methotrexate	1–1.5 g/mE+2/d, day 1
Asparaginase	3,750 U on day 3
*Maintenance chemotherapy*	
Methotrexate	20 mg/mE+2, weekly
6-mercaptopurine	60 mg/mE+2, days 1 to 28
Vindesine	4 mg, day 1
Prednisone	1 mg/kg, days 1 to 7

**Table S2:** Primer and probe sequences of *CSRP2* and *ABL1*

**Primer or probe**	**Sequence (5-’3’)**
*CSRP2* forward primer	GTGATGGCAGGAGCTTCCA
*CSRP2* reverse primer	GCCACTGTTGTGCTATCTAAATTTTT
*CSRP2* probe	FAM-CGCTGCTGCTTTCTCTGCATGGTTT-BHQ
*ABL1* forward primer	CCGCTGACCATCAATAAGGAA
*ABL1* reverse primer	GATGTAGTTGCTTGGGACCCA
*ABL1* probe	FAM-CCATTTTTGGTTTGGGCTTCACACCATT-TAMARA

CSRP2: Cysteine and glycine-rich protein 2.
